# Evaluating surface EMG control of motorized wheelchairs for amyotrophic lateral sclerosis patients

**DOI:** 10.1186/s12984-022-01066-8

**Published:** 2022-08-14

**Authors:** Albert C. Manero, Shea L. McLinden, John Sparkman, Björn Oskarsson

**Affiliations:** 1 Limbitless Solutions, University of Central Florida, 12703 Research Parkway, Suite 100, Orlando, USA; 2 ALS Center of Excellence, Mayo Clinic, 4500 San Pablo Road, Jacksonville, 32224 USA

**Keywords:** Amyotrophic lateral sclerosis, Electromyography, Muscle activation, Wheelchair, Temporalis muscles

## Abstract

**Background:**

This study evaluated a novel control method for patients unable to independently control powered wheelchairs. Patients with amyotrophic lateral sclerosis often require a wheelchair but struggle with sufficient hand dexterity required for joystick control making them a population that needs this type of control method.

**Methods:**

The study employed a novel control mechanism, using electromyography surface sensors applied to temporalis muscles able to measure the myoelectric voltage. Pattern and magnitude control of muscle contraction allowed for steering intention recognition and were used to manipulate their power wheelchair joystick. Four patients ages 51 to 69, two female and two male with amyotrophic lateral sclerosis, conducted Wheelchair Skills Test developed by Dalhousie University and were surveyed on the experience’s Clinical Global Impression of Change.

**Results:**

Findings showed independent steering was capable for patients without hand function and provided recommendations for improved human-machine interface. All patients demonstrated the ability to engage the system, with varying precision, for driving their wheelchair in a controlled environment.

**Conclusions:**

Three patients in the pilot trial reported the highest score of clinical global impression of change, all of whom had lost independent control of their wheelchair joystick. Patient four retained impaired hand dexterity for joystick control and reported negative impression of change, comparatively. Feedback from the study will be leveraged to improve training outcomes.

*Trial registration* Subjects provided signed informed consent according to the Declaration of Helsinki to enter the study that was approved by the Mayo Clinic Institutional Review Board in Rochester, Minnesota. The study is registered on ClinicalTrials.gov under identifier NCT04800926 as of March 14, 2021 retrospectively registered.

## Background

Amyotrophic lateral sclerosis (ALS) is a disease caused by the degeneration of motor neurons in the nervous system leading to decreased mobility [1, 2]. The United States ALS incidence rate is 3.9 cases per 100,000 persons diagnosed yearly [3, 4]. ALS patients lose motor function causing spasticity, lack of precision and loss of muscle contraction [2]. This leads to reduction of independent ambulation where power wheelchairs provide means of retaining mobility, a greater quality of life and increased feelings of independence [5, 6]. Functional impairment of upper extremities eventually affects the ability to control devices [7]. Advances to powered wheelchair controls for patients with limited dexterity and mobility exist on a spectrum from independent control to shared control, where shared control could include pre-program paths and routines to support situation specific driving [8].

Limited progress has been made in the field due to declining mobility during later stages of ALS, presenting challenges in obtaining longitudinal data in addition to the psychological distress from the quantification of decline that participants may experience [9, 10]. Studies demonstrate the decline in finger dexterity as the main cause of the declining satisfaction of controller use, inspiring wheelchair modifications that use electrochemical signals from the mastication muscles. Surface EMG quantifies the activation of the muscle which is shown to correlate to the force of the muscle contraction [11]. Signals captured by the sensor have been used to detect levels of contraction in upper limb muscles and translated into commands for wheelchair control [12].

Before the production of force by a particular muscle, electrical potential is produced which is what electrodes on the surface of skin can measure [13]. The surface EMG is used as opposed to a needle EMG which is more invasive [14]. These signals captured by the sensor have successfully been used to detect varying levels of contraction in upper limb muscles and translated into commands for wheelchair control using electromyography on the temporalis muscles by the authors [12]. Ongoing research developments focused on EMG measurements and controls for powered wheelchairs [15] have been compiled by Kaur [15], who reported a subset of 15 applicable studies, though limited research has been published detailing patients with insufficient hand dexterity. Xu et al. [16] has demonstrated control using a single and double jaw click, collecting signal from the masseter muscle and buccinator muscle, while Moon et al [17] mapped control to the levator scapulae muscle, via bilateral shoulder elevation for alternative ways to provide steering intention for powered wheelchairs.

### Interface and decoupling

ALS patient condition evolves into multiple functional stages with a transient nature. While initial symptoms emerge subtly, the effects to motor neuron connectivity become profound resulting in an inability to volitionally use most muscles [18]. Some cases may present with pronounced effects to either the upper or lower motor neurons resulting in spasticity or atrophy. This presents as abnormalities of (i) a loss of the relative magnitude contraction and (ii) the loss of discretization between rest and contraction (causing a more binary case of rest and clench) [19] in differing proportions. With the advent of assistive mechanical ventilation and power wheelchairs life quantity and often quality can be maintained with a degree of independence despite profound disability. A preserved ability to communicate and independently move through the world is key to sustaining a meaningful life in the face of progressive disability. This electromyographic wheelchair controller is aimed at addressing this need for people who have lost the ability to use current control interfaces, and in extension to become the primary choice of control system.

Similar studies have been conducted with powered wheelchairs for those with limited mobility using EMG to sense muscle usage [20, 21]. Researchers have looked for additional control methods, one of which being control of the chair by a single muscle group of choice which can reduce physical effort and fatigue [22]. Another study also utilized facial muscles whose movement was captured through electromyographic signals to navigate a virtual wheelchair which also uses thresholding to help combat fatigue overtime [23]. These studies show the variety of applications of EMG control of powered medical devices and training opportunities and benefits.

Non-EMG wheelchair interfaces are also available as alternative control methods for wheelchair users, especially those with reduced hand dexterity that limits use of joystick controls. A different potential control interface includes eye gaze interaction as a method of communicating with and controlling a device [24]. One current limitation of this control schema is the variability of lighting conditions which makes it difficult for constant eye detection [25]. Chin controls is also an available control schema but may not be effective for someone with a neuromuscular disease that has progressed beyond their ability to control chin movement [26].

Initial user feedback provided insight into how disease progressions could challenge the use EMG signals from temporalis controls for wheelchair steering. While the system began with bilateral control using two sensor’s inputs on both temporalis muscles, this mode was difficult for some patients to decouple muscle contractions. This complication could be exasperated for patients who may have predominant unilateral facial muscle weakness. For these cases, mode one using multi-gesture, unilateral, magnitude input was utilized. This allowed for unilateral control of the system, through discretizing the relative contraction range into three distinct regions. While this method was reported to be more complex to visualize, it did prove effective to provide steering for the system. These methods of control were chosen because the offer the ability to control any wheelchair via a modification placed on top of the joystick as opposed to modifying any of the hardware or software components of the wheelchair. By not making permanent changes to the wheelchair it preserves the warranty and insurance on the powered device. The two modes for control have unique challenges for training and learning, which has led the investigators to now explore methods for providing visual feedback and simulation through gamified intervention.

ALS patients can train varying muscle contraction signals to outcomes of a mechanical component, in this case is a powered wheelchair. Different signals received from various contraction magnitudes can be uniquely assigned to an output on the device. The device is calibrated based on the measured EMG signal for each individual, as long as there is sufficient capability of intentionally contracting and relaxing the muscle. EMG signals can be calibrated to engage a direction of motion for the wheelchair.

For bilateral control, a short contraction of the left and right mastication muscle groups will correlate with turning left and right respectively, while a short clench on both sides corresponds to forward and a long clench to backwards. In unilateral control, a small magnitude clench will engage left control, medium for the right, and high corresponding to forward.

A bilateral rapid clench, while already in motion, will halt motion and similarly in unilateral control uses a short rapid contraction signal for immediate halting. Both methods will halt motion if there is a loss of signal from the sensors to the device control interface.

An optional control for reverse control can be included, using the same engagement for forward but with a longer clench duration on the order of < 1 s.

This manuscript reports the outcomes of an pilot clinical trial to evaluate the usability of temporalis EMG control for a powered wheelchair for patients with limited hand dexterity.

## Methods

### Patients with amyotrophic lateral sclerosis and study visit

This study recruited patients with an ALS diagnosis by revised el Escorial criteria [27], between ages 18 and 89, and with impairment of hand function limiting the use of a standard joystick control. Subjects provided signed informed consent according to the Declaration of Helsinki to enter the study that was approved by the Mayo Clinic Institutional Review Board in Rochester, Minnesota. Additional exclusion criteria included (i) cognitive impairment prohibiting safe independent mobility is defined by an ALS Cognitive Behavioral Screen (ALS-CBS) score of < 10 or the opinion of the investigator, in addition to (ii) severe loss of facial muscle functionality or control that would preclude EMG electrode efficacy. The four patients who participated in the study were selected due to advanced and slowly progressive ALS. Patient demographics have been summarized and presented in Table 1.

**Table 1:** Patient demographics

Participant	Sex	Age	Disease duration (years)	FRSr
1	F	54	6	9
2	M	69	10	20
3	M	57	3	12
4	F	51	3	9

Patients were evaluated on ALS Functional Rating Scale (FRSr) [28]. The Amyotrophic Lateral Sclerosis Functional Rating Scale (ALSFRS) evaluates the functional status of patients with Amyotrophic Lateral Sclerosis and is used to monitor functional changes in a patient over time [29]. ALS FRSr scores for each patient are documented in Table 1. Participant 1 at the time of enrollment 54 years of age, with a disease duration of 6 years, utilizing continuous ventilation with an FRSr of 9. She remains alive. Participant 2 was 69 years old with a disease duration of 10 years and a FRSr of 20. He was not utilizing ventilatory support and remains alive. Participant 3 was a 57 year old man with onset of disease in his legs 3 years prior to study enrollment. He was utilizing continuous ventilation and had a FRSr score of 12. He remains alive. Participant 4 was a 51 year old woman with a 3 year disease duration, who was utilizing continuous ventilatory support and had an FRSr of 9. She passed from ALS after 5.5 years of symptomatic disease.

Study visits were about 1 hour long and contained set up, training, and evaluation as described in the wheelchair control system subsection. Initial calibration for each wheelchair was complex and took significant time, as the specifics of the chair varied patient to patient substantially. EMG calibration was much faster for following study visits, once an optimum sticker placement was established. Study procedures by visit are outlined in Table 2. For most study visits more time was spent training than optimizing set up.

**Table 2:** Study procedures by visit

Study activity	Visit 1	Visit 2	Optional visit 3
Informed consent	X		
Review of medical history and demographics	X		
Medical history interim		X	X
Complete physical exam	X		
Limited physical exam (inspection of skin)	X	X	X
ALS CBS collected from a previous visit	X		
Concurrent meds collected from last visit	X		
ALSFRS-r collected from a previous visit	X		
Wheelchair safety and other adverse event reporting	Past	X	X
WST (for standard control)	X	X	X
Training course	X	X	X
WST (for novel control)	X	X	X
Global impression of satisfaction CGI-C	X	X	X

### Wheelchair control system

**Fig. 1:**
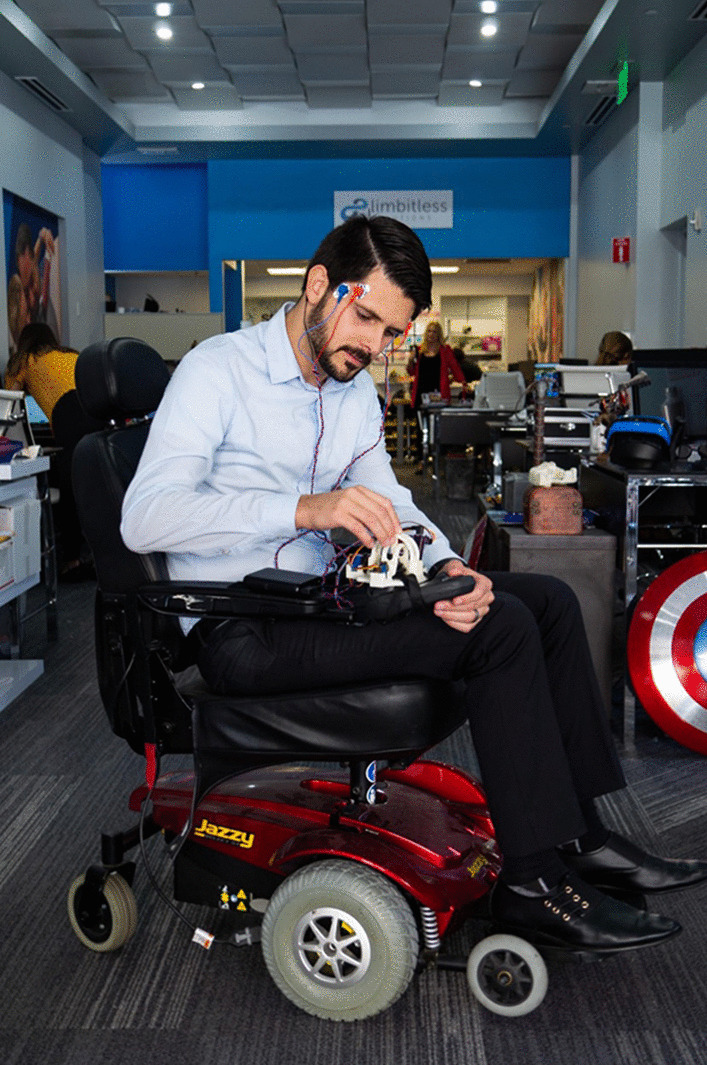
An overview of the EMG control of motorized wheelchair devices. Pictured is a user utilizing electromyography to control the movement of the joystick via the attachment to the wheelchair device

The system employed for the study was designed to support mobility devices that do not rely on hand usage. The control schema is based on electrical signals sent from the contraction of the temporalis muscles, with signals captured by the EMG sensors that can be setup in two modes. Figure 1 shows the novel wheelchair control device in use.

Prior to using one of the modes the device is connected to the mobile app via Bluetooth and is calibrated within the app. The calibration mode allows for account of the signal strength that each user puts out. The calibration mode is also helpful because you can set the threshold for each signal, and beneath that threshold tells the device to disregard noise from their resting position. It also sets the threshold for reaching each discretized level for the mode that they choose. When the device is attached it is important to place it in a location that optimized capturing the movement of the temporalis muscle and additionally that the ground in placed behind the ear, where no muscle movement is recorded. The primary population that this device is intended for has a need for a full time caregiver who would also be trained in the calibration and set up of the device. Calibration can be shifted with the changing capabilities of the user. As the muscle activity declines, which can occur across the day from fatigue or dehydration, the calibration can be changed in the mobile app to reflect new values for each threshold of control.

**Fig. 2:**
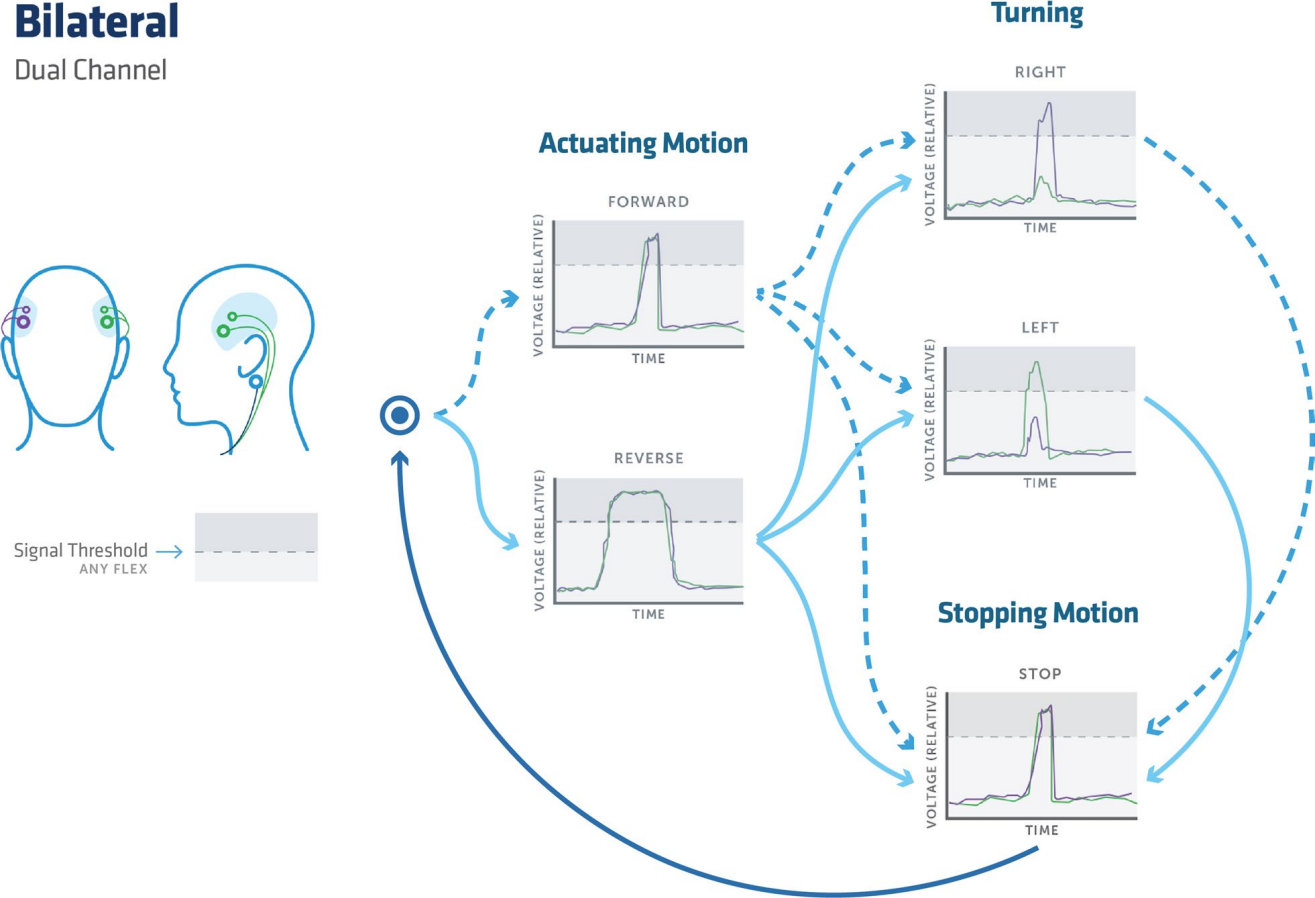
An overview of the bilateral input mode for the control system. The process flow chart begins and follows the users input signal to initiate a forward or reverse motion with a clench of both temporalis muscles with either a short or long contraction for a forward or reverse command respectively. The user can then initiate the stopping function, which is the same input as the forward command, or begin a turning motion while maintaining forward motion. A turning motion is initiated by a contraction of the temporalis muscle on the side of intended motion. If a turning motion is chosen it can be stopped with an additional turning command and maintain forward motion. Or for a complete stop, the aforementioned stop function will arrest all motion. Once the stopping motion has occurred the user can guide themselves through the process flow again. The user can also begin mid-flow with a simple left or right input command without moving in a forward or reverse motion

**Fig. 3:**
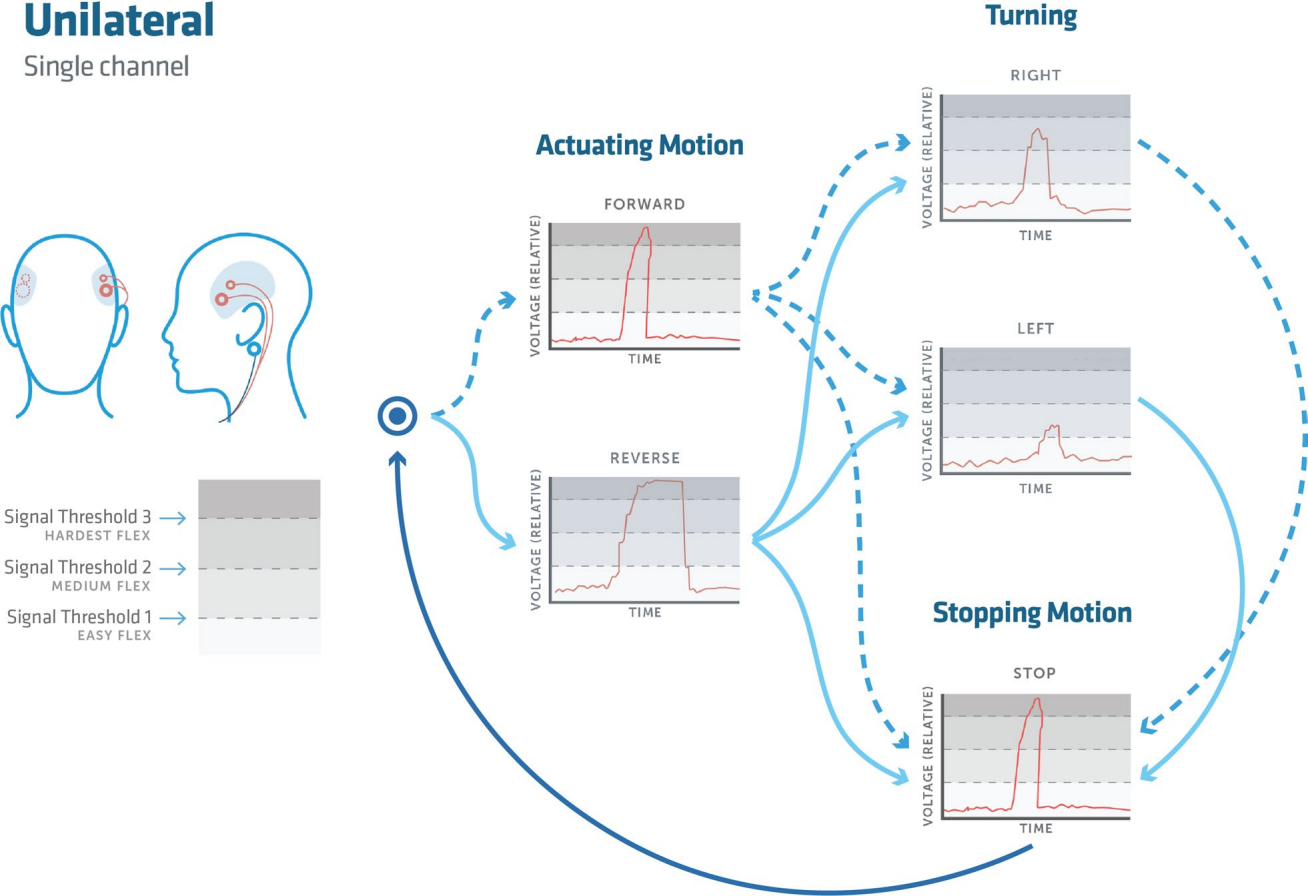
An overview of the unilateral input mode for the control system. The process flow chart begins at the bulls-eye and follows the users input signal to initiate a forward or reverse motion. The forward command is initiated by a hard contraction of a short duration, while the reverse motion is a hard contraction of a long duration. From here the user can then initiate the stopping function, which is the same input as the forward command, or begin a turning motion while maintaining forward motion. A turning motion is chosen it can be stopped with an additional turning command and maintain a forward motion. Or for a complete stop, the aforementioned stop function will arrest all motion. Once the stopping motion has occurred the user can guide themselves through the process flow again. The user also can begin mid-flow with a simple left or right input command without moving in a forward or reverse motion

**Fig. 4:**
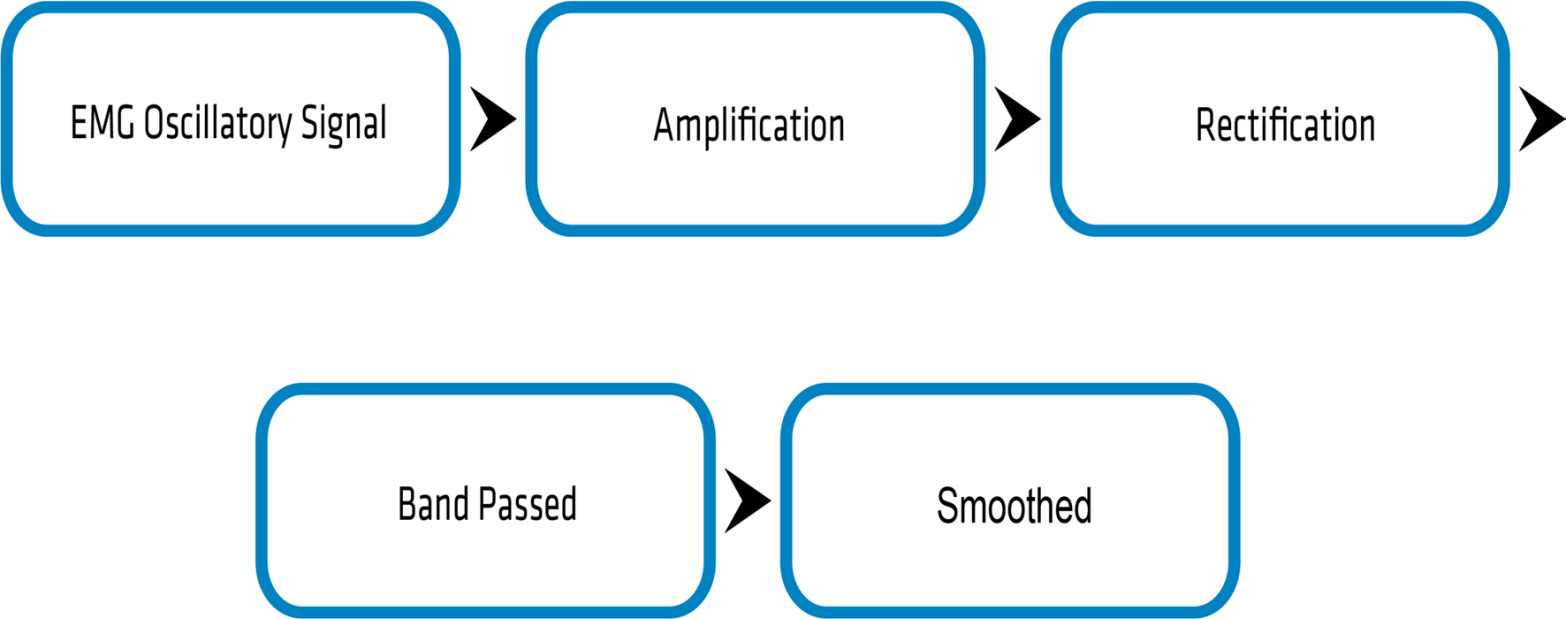
An overview of the signal processing chain. The EMG oscillatory signal input is amplified, rectified, band passed, and then smoothed

Mode one, shown in Fig. 2 demonstrates a bilateral control scheme, where left and right temporalis sets provided a combination of binary inputs. Mode two, shown in Fig. 3 used a unilateral electrode placement that captured relative muscle contraction magnitude signals, with three discrete magnitude zones, labeled as signal thresholds for controlling motion. Unilateral control was employed in response to patients with difficulty decoupling bilateral muscle controls. Any gesture command will remain active until the stopping motion is activated. Left and right steering may be engaged while maintaining forward or reverse, which remains active until a secondary left or right signal returns the control to forward or reverse. Reverse control was initially disabled for two out of the four participants on the first trial and then disabled for all participants in further evaluations for ease of use as they were learning the control schema. A schematic of the control scheme and modes is presented in both Figs. 2 and 3. A breakdown of the signal processing chain is presented in Fig. 4.

A physical control device was devised as a temporary retrofit attachment to most powered wheelchairs. This was done to minimize damage to the chair or its warranty.

#### Bilateral control scheme

Bilateral control mode is defined by its use of dual EMG sensors represented as the purple and green electrodes in Fig. 2. The electrical signal inputs picked up by the electrodes as a result of muscle clenches are shown in the corresponding graphs of Fig. 2. The lateral view shows a third electrode which is represented in blue color. This electrode acts as a ground for the device and is used on both the left and right lateral views.

The lowest magnitude threshold also provides affordances to avoid convolution of the contraction signal with the noise band. As the device was retrofitted for each patient’s chair uniquely, each chair may have different maximum speeds or joystick settings as some chairs will reduce speed while in forward and a left or right turn. Others will do this unintentionally, due to the position of the servo motor may not be set to accommodate the full range of motion for the joystick. In the incident of loss of signal, the wheelchair will come to an immediate halt.

#### Unilateral control scheme

Unilateral control mode is defined by its use of a single EMG sensor controlled by either the left or the right side of the face, shown in Fig. 3. This method of control is most useful for patients with more significant control ability on one lateral half of their face. Figure 3 shows that the electrodes can be placed on the left or the right side of the users face, whichever is found to be more conducive for implementation. In the corresponding lateral image shows a third, represented in blue electrode located behind the ear which acts as a ground for the device just as in the bilateral control method. Magnitude thresholding is used to correlate a voltage output with how hard the user is contracting the muscle group to generate unique outcomes on the chair.

### Assessment methods

This study used a simple open room available at the hospital, partitioned into an unobstructed four by four meter area, designated by the placement of four cones forming a perimeter. Within the zone, participants tested their general wheelchair control, command of direction, and simulated residential obstacle avoidance. The Clinical Global Impressions of Change provided an instrument to quantify the progress of current wheel chair control to the new system, along with progress assessment from the multiple sessions [30, 31]. CGI-C scoring reported from one (very much improved) through to seven (very much worse), relative to the patient’s baseline impression of their current equipment and condition.

The Dalhousie University wheelchair skills test was employed to evaluate the patients’ mobility and control using their power wheelchair with the electromyographic control scheme [32, 33]. The Dalhousie University wheelchair skills test was used to measure the ability to, and confidence in movement of the wheelchair forwards, backwards and turning. The wheelchair skills test dictated a specific protocol for movement of the wheelchair device by the participants. Not every question was evaluated as applicable to the study, due to limitations in testing and the scope of the control inputs, where the control system only interacts with steering control on the wheelchair. For evaluation of the wheelchair skills, the scoring for capacity is on a zero to two integer scale, where a zero is a failure to complete the assessment, one is considered passing with difficulty, and two is identified as passing. Due to the limited questions able to be evaluated on the questionnaire for this study, the survey results have been reported in a modified capacity to capture the steering control capabilities and relative difficulty reported for each patient.

## Results

Table 3 presents the modified wheelchair skills test reporting for each patient, focused on their ability to engage the electromyographic steering control system. The wheelchair skills test is designed to be adapted to any wheelchair model. For this study, the results show participant’s ability to move forwards, backwards, turning and their ability to operate in both directions. The reported results showed that the control system was feasible and effective in the scope of the testing.

For patients without hand or foot steering controls, the wheelchair device can be used for independent control during the course testing.

**Table 3:** Wheelchair motion study evaluation from Wheelchair Skills Test (WST) [32, 33] with degree of difficulty reported

Patient	Evaluation 1		Evaluation 2		Evaluation 3^d^	
alpha	*Forward*	Yes^b^	*Forward*	Yes^b^	*Forward*	Yes^b^
	*Backwards*	Yes^a^	*Backwards*	Yes^a^	*Backwards*	Yes^b^
	*Turning*	Yes^a^	*Turning*	Yes^a^	*Turning*	Yes^b^
	*Direction*	Both	*Direction*	Both	*Direction*	Both
beta	*Forward*	Yes^c^	*Forward*	Yes^a^		
	*Backwards*	No	*Backwards*	Yes^a^		
	*Turning*	Yes^c^	*Turning*	Yes^a^		
	*Direction*	Both	*Direction*	Both^a^		
gamma	*Forward*	No	*Forward*	Yes^a^		
	*Backwards*	No	*Backwards*	No		
	*Turning*	No	*Turning*	Yes		
	*Direction*	No	*Direction*	Both		
delta	*Forward*	No	*Forward*	Yes^a^		
	*Backwards*	No	*Backwards*	No		
	*Turning*	No	*Turning*	Yes		
	*Direction*	N/A	*Direction*	Both		

^a^Very well

^b^Yes

^c^Yes with difficulty

^d^Participants were asked to complete a minimum of two or a maximum of three study visits

The patient and caregiver surveys of the impression of change show nearly all highly favorable scores, due to the system’s ability to provide a level of independence. The Clinical Global Impressions of Change is a standardized measure to determine treatment efficacy [31]. This control was previously unavailable to the patient due to their condition and present wheelchair control methods which were only an attendant joystick on the rear of the chair. The results are presented in Table 4 below. Three patients indicated the highest positive score for the impression of change. Patient beta scored the test of the electromyographic control system as unfavorable relative to the foot control, which enabled their baseline control to be superior to the electromyographic control system that they had been utilizing with for over a year. However, the pilot data demonstrates that for patients with limited independent control of their wheelchair steering, the system performs favorably.

**Table 4:** Clinical global impression of change (CGIC) survey [31] evaluation

Patient	Evaluation 1	Evaluation 2	Evaluation 3^a^
alpha	1	1	1
beta	7	7	
gamma	N/A	1	
delta	1	1	

^a^Participants were asked to complete a minimum of two or a maximum of three study visits

## Discussion

Patients showed an increase in control over the EMG system, as indicated by favorable scores of the impression of change. The advantages of this novel control device open up the opportunity for independent control of wheelchair movement for patients with loss of dexterity, especially in populations that experience neurodegenerative decline. For patients who experience no independent mobility, the device was well received as a new accessible method in a controlled environment.

### Training and evaluation considerations

Kaur [15], who reported a subset of 15 applicable studies, though limited research has been published detailing patients with insufficient hand dexterity. Xu et al. [16] focused testing for participants with no accessibility limitations, collecting signal for steering using the masseter muscle and buccinator muscle. An emphasis on virtual training, by maneuvering through a figure 8 course, and demonstrated lap time reduction from gained experience. These virtual training methods may provide future comparison points, independent of the muscle selected for signal acquisition.

While the data reported herein this manuscript does not include timing data for comparison, it is of interest forward to have standardized driving time trials to better understand training efficacy.

Moon et al. [17] study’s extension to a controlled, physical test environment was conducted virtually [17], with participants without accessibility limitations, and demonstrated the feasibility of control and training through a set of virtual obstacle course with comparison to users with a keyboard input interface. These virtual training studies, with a comparison to hand dexterity input control, can lead to an improved understanding of the training improvement and overall usability comparisons.

This manuscript herein uniquely reported Clinical Global Impressions of Change survey results from four patients with mobility and dexterity limitations due to ALS and provides a unique perspective of the perceived significance from independent wheelchair mobility. As future virtual training specific studies are being planned, developing a testing strategy that compares across various types of input methods will lead to more robust reporting.

While there is significant potential for adding a new mobility tool for patients, where appropriate during the syndrome’s progression, additional complexity does have drawbacks. Learning a new system for wheelchair control can be an additional burden to patients and caregivers and even add frustration. Due to the complexity of training, the reverse feature, a time dependent input, was turned off. The patients showed marked improvement in their ability to engage the control system. This viewpoint has inspired continued development and improvement of the training system to increase the ease of control by the patients with the device and to support the caregiver for setup and calibration when necessary.

Additional training opportunities may also give a chance to practice placement of EMG electrodes and calibrating the device for both patients and caregivers, which had not the complexity of setup. While the placement of the EMG sensors has some affordances for variation, the more often the caregiver or patient is participating may lead to recognizing physical landmarks for placement of the sensors. During testing it was found that many powered wheelchairs have limitations in performing small precision maneuvers at low speeds on carpeted surfaces, making this assessment challenging.

Kaur [15] has aggregated the different sensor configurations from the field of research’s manuscript. Applying the sensors to the face muscles may provide patients with a wider range of ailments a sufficient human machine interface signal, as these muscles may be less likely to be compromised by a variety of pathological conditions. Though, as ALS symptoms and progressions may vary from patient to patient, challenges with facial muscle controls may persist and result in difficulty for some patients to decouple muscle contractions or to relax one side of the face. This complication could be exasperated for patients who may have predominant unilateral facial muscle weakness. Other patients may exhibit challenges with discretizing muscle contraction beyond a near binary rest or maximum. These variations catalyzed the development for both the unilateral and bilateral control schemes, which may reduce the time and ability required to accurately utilize the system.

The data collected and feedback from patients and caregivers will be used to advance a new visual training system and to scale for a larger population clinical trial. Accelerating training by restructuring the in-person assessment to include simulation training only at the first visit, with the option to continue training at home, may improve the level of comfort with the system. Adjustments to the testing environment including flooring choices and realistic room obstacles will allow for a better evaluation of the readiness of the system for at home patient use. Additional timed measurements for course completion, with respect to training progress, may provide additional opportunities for improving control schemes and training as discussed in the literature [16, 17].

## Conclusions

The novel control equipment and input schemes demonstrated effectiveness and enabled patients to move their wheelchair independently in the controlled environment. Three patients in the pilot trial reported the highest score of clinical global impression of change, all of whom had previously lost independent control of their wheelchair joystick. Patient four retained impaired hand dexterity for joystick control and reported a negative impression of change, comparatively. All patients completed the Wheelchair Skills Test, for applicable categories, and demonstrated independent control of the wheelchair via their temporalis muscles EMG signaling.

This study demonstrated the feasibility of providing complex controls for mobility equipment through minimally invasive temporalis mounted sensors, with additional work proposed to increase training outcomes in a larger scale trial. By demonstrating the successful implementation of muscles on the face, patient groups with severe mobility limitations may benefit from independent control of their rehabilitative equipment. The use of the temporalis muscles may translate the technology for high spinal cord injuries, quadriplegia cases, or other neuromuscular disorders that traditionally have limited options for self-directed mobility.

## Data Availability

The authors confirm that the data supporting the findings of this study are available within the article [and/or] as Additional file by request.

## Abbreviations

ALS: Amyotrophic lateral sclerosis
EMG: Electromyography
ALS-CBS: Cognitive Behavioral Screen
CGI-C: Clinical Global Impressions of Change
ALSFRS-r: Revised Amyotrophic Lateral Sclerosis Functional Rating Scale

## References

[1] Oskarsson B, Gendron TF, Staff NP. Amyotrophic lateral sclerosis: an update for 2018. In: Mayo clinic proceedings, vol. 93. Elsevier; 2018. p. 1617–28. 10.1016/j.mayocp.2018.04.007 30401437

[2] Wijesekera LC, Nigel Leigh P (2009). Amyotrophic lateral sclerosis. Orphanet J Rare Dis.

[3] Arthur KC, Calvo A, Price TR, Geiger JT, Chio A, Traynor BJ (2016). Projected increase in amyotrophic lateral sclerosis from 2015 to 2040. Nat Commun.

[4] Mehta P, Kaye W, Bryan L, Larson T, Copeland T, Wu J, Muravov O, Horton K (2016). Prevalence of amyotrophic lateral sclerosis-united states, 2012–2013. Morb Mortal Wkly Rep Surveill Summ.

[5] Bello-Haas VD, Kloos AD, Mitsumoto H (1998). Physical therapy for a patient through six stages of amyotrophic lateral sclerosis. Phys Ther.

[6] Meyrick A. Powered wheelchair provision for adults diagnosed with rapidly deteriorating MND. Posture and Mobility Group. 2016;2.

[7] Oskarsson B, Joyce NC, De Bie E, Nicorici A, Bajcsy R, Kurillo G, Han JJ (2016). Upper extremity 3-dimensional reachable workspace assessment in amyotrophic lateral sclerosis by Kinect sensor. Muscle Nerve.

[8] Cowan RE, Fregly BJ, Boninger ML, Chan L, Rodgers MM, Reinkensmeyer DJ (2012). Recent trends in assistive technology for mobility. J Neuroeng Rehabilitat.

[9] Andres PL, Slavin MD, Jette DU, Munsat TL (1997). Lower extremity muscle strength measures in patients with amyotrophic lateral sclerosis. Neurol Rep.

[10] Arts IM, Overeem S, Pillen S, Schelhaas HJ, Zwarts MJ (2011). Muscle changes in amyotrophic lateral sclerosis: a longitudinal ultrasonography study. Clin Neurophysiol.

[11] Disselhorst-Klug C, Schmitz-Rode T, Rau G (2009). Surface electromyography and muscle force: limits in sEMG-force relationship and new approaches for applications. Clin Biomech.

[12] Manero A, Oskarsson B, Sparkman J, Smith PA, Dombrowski M, Peddinti M, Rodriguez A, Vila J, Jones B. Xavier electromyographic wheelchair control and virtual training. In: International conference on human–computer interaction. Springer; 2019. p. 133–42.

[13] Day S (2002). Important factors in surface EMG measurement.

[14] Merletti R, Parker PJ (2004). Electromyography: physiology, engineering, and non-invasive applications.

[15] Kaur A (2021). Wheelchair control for disabled patients using EMG/EOG based human machine interface: a review. J Med Eng Technol.

[16] Xu X, Zhang Y, Luo Y, Chen D (2013). Robust bio-signal based control of an intelligent wheelchair. Robotics.

[17] Moon I, Lee M, Chu J, Mun M. Wearable EMG-based HCI for electric-powered wheelchair users with motor disabilities. In: Proceedings of the 2005 IEEE international conference on robotics and automation. 2005. p. 2649–54.

[18] Schmidt ER, Pasterkamp RJ, van den Berg LH (2009). Axon guidance proteins: novel therapeutic targets for ALS?. Progr Neurobiol.

[19] Bruijn LI, Miller TM, Cleveland DW (2004). Unraveling the mechanisms involved in motor neuron degeneration in ALS. Annu Rev Neurosci.

[20] Lung C-W, Chen C-L, Jan Y-K, Chao L-F, Chen W-F, Liau B-Y. Activation sequence patterns of forearm muscles for driving a power wheelchair. In: International conference on applied human factors and ergonomics. Springer; 2017. p. 141.

[21] Lung C-W, Cheng T-Y, Jan Y-K, Chen H-C, Liau B-Y (2016). Electromyographic assessments of muscle activation patterns during driving a power wheelchair. Advances in physical ergonomics and human factors.

[22] Felzer T, Freisleben B. Hawcos: the “hands-free” wheelchair control system. In: Proceedings of the fifth international ACM conference on assistive technologies. 2002. p. 127–34 .

[23] Silva A, Morère Y, Naves E, De Sa A, Soares A. Virtual electric wheelchair controlled by electromyographic signals. In: 2013 ISSNIP biosignals and biorobotics conference: biosignals and robotics for better and safer living (BRC). IEEE; 2013. p. 1–5.

[24] Araujo JM, Zhang G, Hansen JPP, Puthusserypady S. Exploring eye-gaze wheelchair control. In: ACM symposium on eye tracking research and applications. 2020. p. 1–8.

[25] Bazrafkan S, Kar A, Costache C (2015). Eye gaze for consumer electronics: controlling and commanding intelligent systems. IEEE Consum Electron Mag.

[26] Puanhvuan D, Khemmachotikun S, Wechakarn P, Wijarn B, Wongsawat Y (2017). Navigation-synchronized multimodal control wheelchair from brain to alternative assistive technologies for persons with severe disabilities. Cogn Neurodyn.

[27] Brooks BR, Miller RG, Swash M, Munsat TL (2000). El Escorial revisited: revised criteria for the diagnosis of amyotrophic lateral sclerosis. Amyotroph Lateral Scler Other Motor Neuron Disord.

[28] ALS CNTF Treatment study (ACTS) phase I–II study group (1996). The amyotrophic lateral sclerosis functional rating scale. Assessment of activities of daily living in patients with amyotrophic lateral sclerosis. Arch Neurol.

[29] Coco DL, Marchese S, La Bella V, Piccoli T, Coco AL (2007). The amyotrophic lateral sclerosis functional rating scale predicts survival time in amyotrophic lateral sclerosis patients on invasive mechanical ventilation. Chest.

[30] Busner J, Targum SD (2007). The clinical global impressions scale: applying a research tool in clinical practice. Psychiatry.

[31] Guy W (1976). ECDEU assessment manual for psychopharmacology.

[32] Kirby RL, Smith C, Parker K, McAllister M, Boyce J, Rushton PW, Routhier F, Best KL, Brandt A (2015). Wheelchair skills program manual 4.0.

[33] Kirby RL, Rushton PW, Smith C, Routhier F, Best KL, Cowan R, Giesbrecht E, Koontz A, MacKenzie D, Mortenson B (2019). Wheelchair skills program manual 5.0.

